# Mining and Characterization of Carbohydrate-Active Enzymes in MAGs from Animal Gut Microbes

**DOI:** 10.3390/molecules31213980

**Published:** 2026-11-04

**Authors:** Lin Han, Ke Tan, Jinming Yu, Wei Ni, Kaiping Liu, Jiangjiao Qi, Shengwei Hu, Xiaoyue Li, Cunyuan Li

**Affiliations:** 1 College of Life Sciences, Shihezi University, Shihezi 832003, China; 2 College of Grassland Science, Xinjiang Agricultural University, Urumqi 830052, China

**Keywords:** gut MAGs, PULs, novel CAZymes reservoir, molecular docking, prokaryotic expression

## Abstract

Background: CAZymes mediate carbohydrate catabolism in animals and are critical for supporting animal growth, development, metabolism and production performance. This study aimed to explore the gut microbial CAZyme repertoire of humans and multiple animal species and identify novel and efficient CAZymes. Methods: A total of 100,090 MAGs from humans and 10 animal species were collected for CAZyme annotation. PULpy was used to annotates PULs encoded by the *Bacteroides*. DeepFRI annotation combined with molecular docking was used to screen novel CAZymes. Prokaryotic expression was further performed to obtain the target enzyme and verify its substrate-degrading activity. Results: We annotated 8,747,365 known CAZymes spanning 613 carbohydrate enzyme families, covering 70.5% of all known CAZyme families. A novel multifunctional CAZyme, *BfLac15*, was identified, which exhibited 24.7% sequence-related similarity. Functional validation confirmed that *BfLac15* can degrade both xylan and β-glucan. Conclusions: The constructed large gut microbial CAZyme library expands our knowledge of CAZymes encoded by the animal gut microbiota and provides resources to promote the development and application of CAZymes.

## 1. Introduction

Carbohydrates are the primary source of energy for animals. They play a crucial role in maintaining animal growth and development, metabolic processes, and production performance [1,2]. However, only monosaccharides and a small proportion of disaccharides can be directly absorbed by animals. Polysaccharides are high-molecular-weight carbohydrates formed by the linkage of multiple monosaccharide molecules through glycosidic bonds. Polysaccharides consist of monosaccharide units linked via glycosidic bonds [3]. Common polysaccharides include starch, cellulose, and glycogen, which have important physiological functions in organisms, such as energy storage, providing energy, and constituting cell structures [4,5,6,7]. Cellulose, a representative recalcitrant polysaccharide, is resistant to enzymatic hydrolysis owing to its highly ordered, tightly packed crystalline structure [8]. The importance of converting complex polysaccharides into usable oligosaccharides or monosaccharides makes exploring and modifying efficient carbohydrate-active enzymes (CAZymes) a popular research topic [9,10]. Novel high-performance CAZymes also hold great promise for advancing biorefinery pipelines and sustainable biochemical manufacturing [11].

The animal gut harbors dense microbial communities specialized in carbohydrate catabolism, which depolymerize complex polysaccharides into monosaccharides available for host absorption and utilization [12,13]. Polysaccharide utilization loci (PULs), gene clusters dedicated to polysaccharide degradation and metabolism, are widely distributed among *Bacteroides*. Individual PULs encode an array of functionally coupled proteins, including cell-surface glycan-binding proteins, outer-membrane oligosaccharide transporters, CAZymes, and transcriptional regulators [14]. As a key group of intestinal glycan-degrading bacteria, *Bacteroides* deploy the coordinated action of multiple PUL-encoded CAZymes to recognize, capture, and depolymerize glycan chains [15], and numerous novel CAZymes with divergent substrate specificities have been identified within its PUL repertories [16]. Unlike this PUL-centered degradation paradigm, members of the genus *Ruminococcus* adopt structurally and functionally distinct carbohydrate-degrading machineries [17]. Canonical *Bacteroides* PULs are co-regulated gene clusters marked by the signature SusC–SusD pair, consisting of a TonB-dependent transporter and a glycan-binding lipoprotein that jointly mediate surface glycan capture and oligosaccharide import across the outer membrane [11]. Their encoded CAZyme repertoires cover a broad spectrum of glycoside hydrolases (GHs), polysaccharide lyases (PLs), carbohydrate esterases (CEs) and carbohydrate-binding modules (CBMs), supporting the degradation of diverse soluble and particulate glycans including host mucin, xylan, pectin and algal polysaccharides; these loci are commonly controlled by hybrid two-component systems (HTCS) or anti-σ factor regulatory circuits, and differential expansion of PULs is recognized as a core strategy enabling niche partitioning among intestinal *Bacteroides* species [12]. In contrast, characterized carbohydrate-degrading systems in *Ruminococcus* lack typical PUL architecture. Instead, this genus assembles secreted, extracellular supramolecular multi-enzyme complexes such as cellulosomes in *R. flavefaciens* and *R. albus*, and amylosomes specialized for resistant starch breakdown in *R. bromii* [13]. These complexes depend on cohesin–dockerin interactions to anchor catalytic CAZymes onto central scaffoldin proteins, concentrating enzymes locally to attack recalcitrant particulate substrates like crystalline cellulose or resistant starch [14]. Their CAZyme profiles are relatively specialized, dominated by starch-active GH13 or cellulose-active GH9/GH48 families, with fewer pectin- or mucin-targeting PLs and CEs; the regulation of these assembly components is substrate-responsive but does not follow the SusC/SusD-centered PUL regulatory framework of *Bacteroides* [15]. Collectively, animal gut microbiota constitutes an enormous, largely underexplored reservoir for mining novel CAZymes.

Here, we performed a bioinformatic mining of CAZyme homologs encoded by gut microbial communities from 11 animal hosts and identified 8,747,365 putative CAZymes. Among these candidates, 90,286 were affiliated to *Bacteroides*. We selected one putative CAZyme, *BfLac15*, encoded by *Bacteroides ovatus* (Vatanent_2016_SRR4408032_bin.1), for in vitro functional validation. Using heterologous prokaryotic expression, protein purification and in vitro enzyme activity assays, we confirmed that *BfLac15* is a novel multifunctional CAZyme capable of hydrolyzing both xylan and β-glucan. This work expands the available CAZyme candidate library, improves our understanding of glycan-degrading potential within animal gut microbiomes, and provides a promising enzyme template for subsequent engineering and biotechnological exploitation.

## 2. Results

### 2.1. Analysis of CAZymes in the Animal Gut

**Figure 1:**
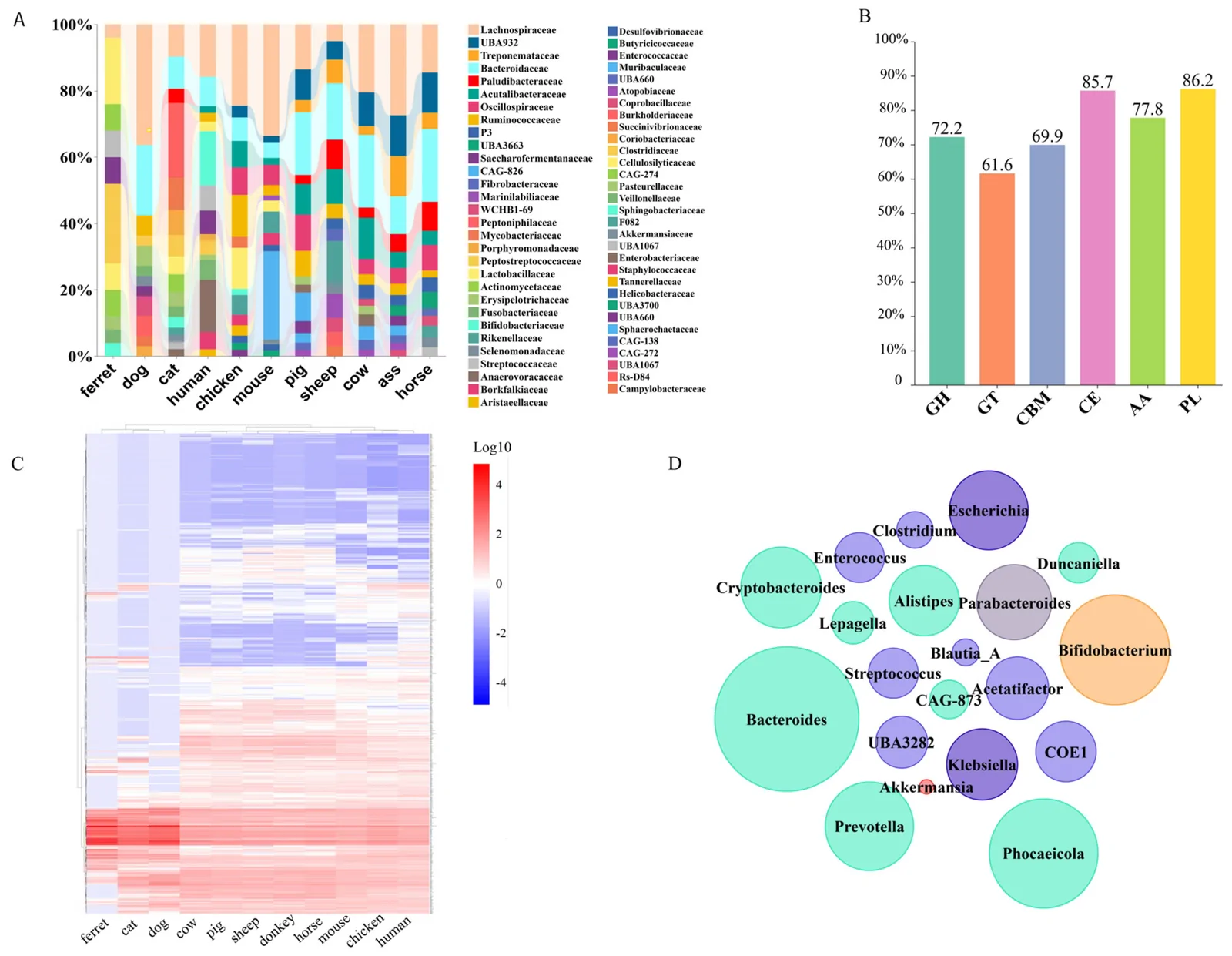
The classification of gut microorganisms and the distribution of CAZymes in different animals. (**A**) Classification of gut microorganisms at the genus level for different animals. The legend on the right represents the genus names. The horizontal axis shows different animal sources, and the animals are sorted according to their feeding habits. The vertical axis represents the proportion of different bacterial genera. (**B**) The coverage of the CAZyme families encoded by the collected intestinal microbial genomes among all known CAZyme families. The x-axis represents different CAZymes families, and the y-axis shows the proportion of the CAZymes families we have annotated among all known CAZymes families. (**C**) The distribution of CAZyme families encoded by MAGs from different animal sources. The horizontal axis represents the clustering of CAZymes of different species, while the vertical axis represents the 613 carbohydrate enzyme families that we have annotated (the redder the color, the greater the number of CAZymes in this family; conversely, the less red the color, the fewer CAZymes there are). (**D**) The top 20 genera with the highest content of CAZymes. The larger the circle, the greater the quantity.

To provide an overview of the CAZymes present in the gut of different hosts, a total of 100,090 MAGs were collected, covering the intestinal tracts of 11 reported animal species. These MAGs were annotated for species classification. A total of 373 archaeal MAGs belonging to 69 genera were obtained. The most common genus of archaea was *Methanocorpusculum*, consisting mainly of methane-producing archaea. Additionally, 99,717 bacteria MAGs were obtained, belonging to 58 classes, 135 orders, 310 families, 1968 genera and 8782 species (Supplementary Table S1). At the genus level, the top five categories were *Bifidobacterium*, *Alistipes*, *Escherichia*, *Streptococcus* and *Bacteroides*. Of these, *Bifidobacterium*, *Alistipes* and *Bacteroides* are primarily probiotic, with both *Alistipes* and *Bacteroides* belonging to the Bacteroidota (Figure 1A).

CAZyme annotation was performed on all MAGs for these 11 species; a total of 8,747,365 CAZymes were annotated. These covered 613 CAZyme families, of which the GH family contained 346, accounting for 72.2% of known CAZyme families (479 GH families). Additionally, there were 85 GT families (61.6%) and 73 CBM families (69.9%). There were 14 AA families (77.8%), 18 CE families (85.7%), and 75 PL families (86.2%) (Figure 1B). Of these, 159 families were shared among the 11 hosts. GHs were found to have the highest proportion in the gut of humans and horses, GTs in mice gut, and CBM in ferret gut (Figure S1A). The GT2_Glycos_trans_2 family was found to be the most abundant among all species (Figure S1B). To further understand the differences in CAZymes in the gut of these 11 species of animals, we analyzed all the CAZymes from different animal sources and found that the carnivorous animals, cat, dog and ferret, were clustered together, while the herbivorous animals, pig, sheep, cow, ass and horse, were grouped together, and the omnivorous animals, mouse, chicken and human, were also grouped together (Figure 1C). In addition, we also found that the six families, GH124, GH16_25, GH45, GH45_1, GH54 and GH5_52, were only found in herbivorous animals, and each herbivorous animal had them. The presence of these carbohydrate enzyme families greatly promotes the digestion and absorption of forage by herbivorous animals. In omnivorous species, the GH62, GH43_23 and GH150 families were discovered, which herbivorous animals and carnivorous animals do not have.

In these samples, the quantity of CAZymes found in the intestines of each species of animal was averaged. Interestingly, the number of CAZymes encoded by the gut microbiota of humans is the highest, with an average of 100.72 per metagenome. This is followed by mice, with an average of 93.88. Donkeys, horses, and cows have an average of over 90, while cats have the lowest average number (only 57.28) (Figure S1C, Supplementary Table S2). Despite being omnivores, humans have a higher average number of CAZymes than ruminants, which feed primarily on grass. Human gut microbes encode 545 carbohydrate enzyme families (Figure S1D), ranking second. Chickens encode the largest number of carbohydrate enzyme families, but their average number of CAZymes is only 69.3, which is much lower than that of humans.

To investigate the ability of different bacteria to degrade carbohydrates, gut microbes were classified at the genus level and the number of CAZymes they encoded was counted. The 20 genera encoding the most CAZymes were extracted. Surprisingly, nine of these genera belonged to the Bacteroidota and seven to the Bacillota. *Bacteroides* encoded the most CAZymes (Figure 1D), significantly more than *Phocaeicola*, which ranked second. Therefore, in the following sections, we will focus on the CAZymes encoded by the *Bacteroides* genus.

### 2.2. Bacteroides Was the Strongest Genus Encoding CAZymes

**Figure 2:**
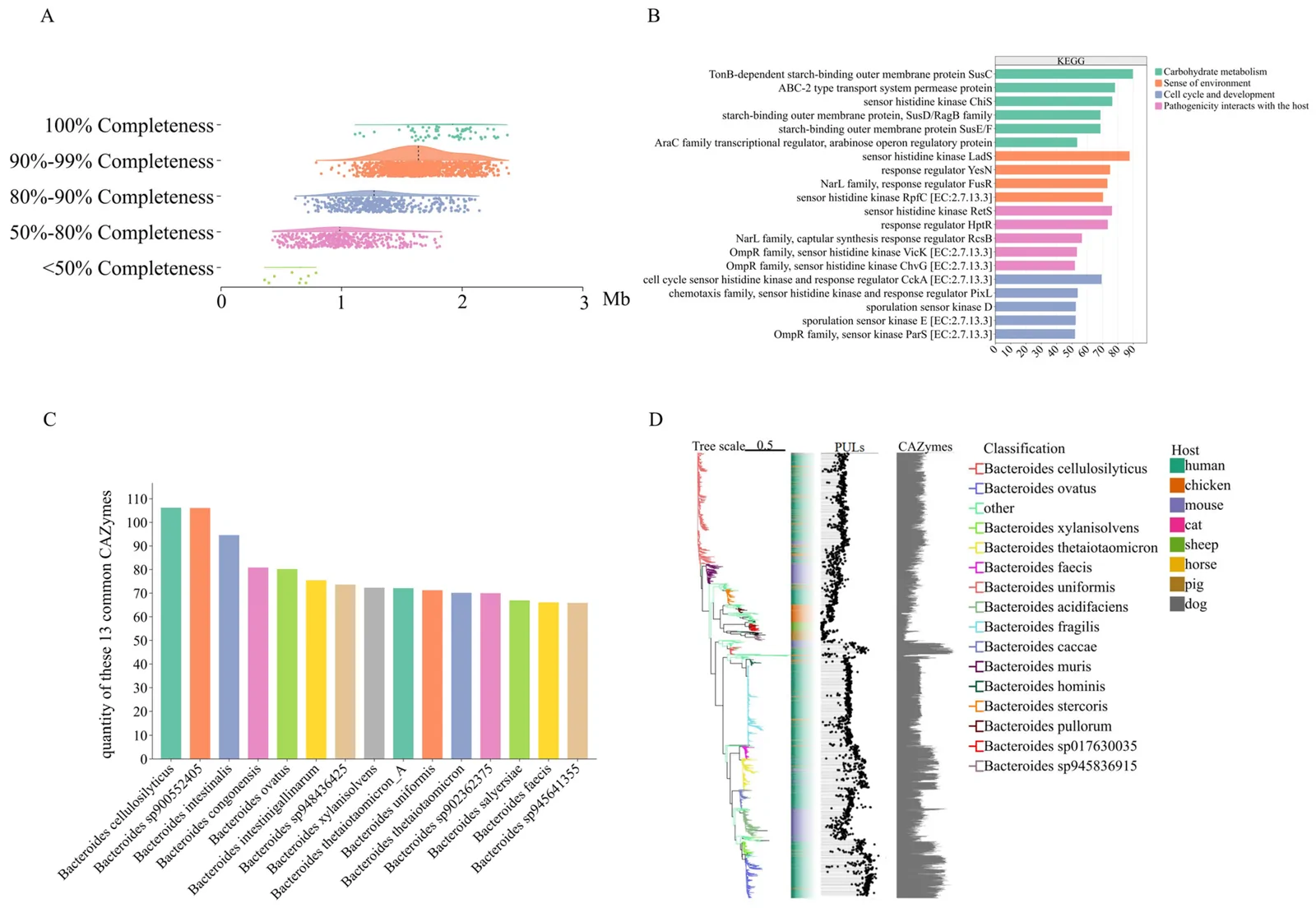
Quality assessment of the genome of the *Bacteroides* genus and its strong ability to synthesize CAZymes. (**A**) Quality assessment and length distribution of the MAGs of the *Bacteroides* genus. The horizontal axis represents the length of the genome, while the vertical axis represents the completeness of the genome. (**B**) Annotation of the signaling pathways of *Bacteroides* species. (**C**) The top 15 bacterial species from the *Bacteroides* genus that encode the highest number of the 13 common CAZymes. The horizontal axis represents the species names, while the vertical axis represents the total quantity of these 13 common CAZymes. (**D**) The phylogenetic tree of *Bacteroides* species and the number of genes encoding PULs and CAZymes. The first column represents the evolutionary tree of the *Bacteroides* and its host. The PULs column indicates the number of PULs encoded by the genome. The CAZymes column shows the number of CAZymes encoded by the genome.

A total of 2228 *Bacteroides* strains were collected from the guts of these 11 hosts. Of these, 1496 strains (65%) were derived from humans. The quality of these *Bacteroides* was assessed using CheckM, revealing that all the genomes had less than 5% contamination, including 67 complete genomes. A total of 2219 medium–high-quality MAGs were identified, accounting for 99.6% of the total. In contrast, there were only nine low-quality MAGs (Figure 2A). Moreover, the genomes of *Bacteroides* are extremely large. The largest genome is single-MIKI_Tac401_18_Tac401co.22.fna (7.2 MB), encoding 6551 proteins, 545 of which are CAZymes (Supplementary Table S3).

All *Bacteroides* species were subjected to KEGG prediction using Kofamscan. The protein with the most annotations was found to be the SusC protein. On average, each genome contained 89.9 SusC proteins (Figure 2B). Additionally, numerous pathways related to CAZymes were identified, including SusE and SusF. This further indicates that the *Bacteroides* genus has a strong ability to degrade carbohydrates. Some common CAZymes were statistically analyzed, including starch phosphorylase (GT35), glycogen synthase (GT3 and GT5), cetanyl xylan esterase (CE6), invertase (GH32), α-amylase (GH13), β-amylase (GH14), endoglucanase (GH5 and GH6), chitinase (GH18 and GH19), pectate lyase (PL1 and PL2), endoxylanase (GH10 and GH11), alginate lyase (PL5 PL6 and PL7), and β-glucosidase (GH1 and GH3) (Supplementary Table S4). *Bacteroides cellulolytic* was found to possess the most of these CAZymes, with an average of 106.1 per genome (Figure 2C), followed by *Bacteroides sp900552405* with 106. Among the five most common species of *Bacteroides bacteria*, *Bacteroides ovatus* encodes 80.1 CAZymes and is also the third most abundant bacterium in the genus.

Phylogenetic analysis revealed that the *Bacteroides* genus, which originated in the human gut, is widely distributed throughout the whole evolutionary tree. This may be related to its large number of MAGs (Figure 2D). The MAGs of the mouse, pig and sheep genomes were clustered. However, it is important to note that *Bacteroides* species from chickens are highly similar to those from humans and are scattered throughout the evolutionary tree. Nevertheless, the average number of CAZymes in the *Bacteroides* species from chickens was only 231, compared to 318 in human *Bacteroides* species, an average difference of 87 CAZymes per species (Supplementary Table S5). The most abundant in the chicken gut, *Bacteroides uniformis*, *Bacteroides pullorum*, and *Bacteroides fragilis*, all encode fewer CAZymes and PULs. In the human gut are *Bacteroides uniformis*, *Bacteroides fragilis*, and *Bacteroides ovatus*, among which *Bacteroides ovatus* has a super-strong coding capacity for PULs and CAZymes.

### 2.3. Mining of the Potential CAZymes Library in Bacteroides

To discover new CAZymes, we annotated the PULs of 2228 *Bacteroides* strains, resulting in a total of 114,047 PULs being identified (Supplementary Table S6). These PULs encoded a total of 229,630 unknown proteins, accounting for 35.4% of the annotated CAZymes. It is worth noting that six low-quality and medium-quality *Bacteroides* MAGs may not have had their PULs annotated due to incomplete genomes. These unknown proteins were annotated using the run_dbcan2 command, which revealed 30,036 protein sequences labeled as CAZymes. This represents 13.1% of the unknown proteins. A total of 185,715 unknown proteins were retained by removing 13,879 protein sequences that were too short in length. In order to remove non-CAZymes, an AI model named DeepFRI was used to conduct functional predictions on 185,715 unknown proteins. From these, enzymes that were annotated as related to CAZymes were selected, totaling 90,286 (Supplementary Table S7). Therefore, we speculate that among these 90,286 proteins, there must be a large number of unknown novel and highly efficient CAZymes.

**Figure 3:**
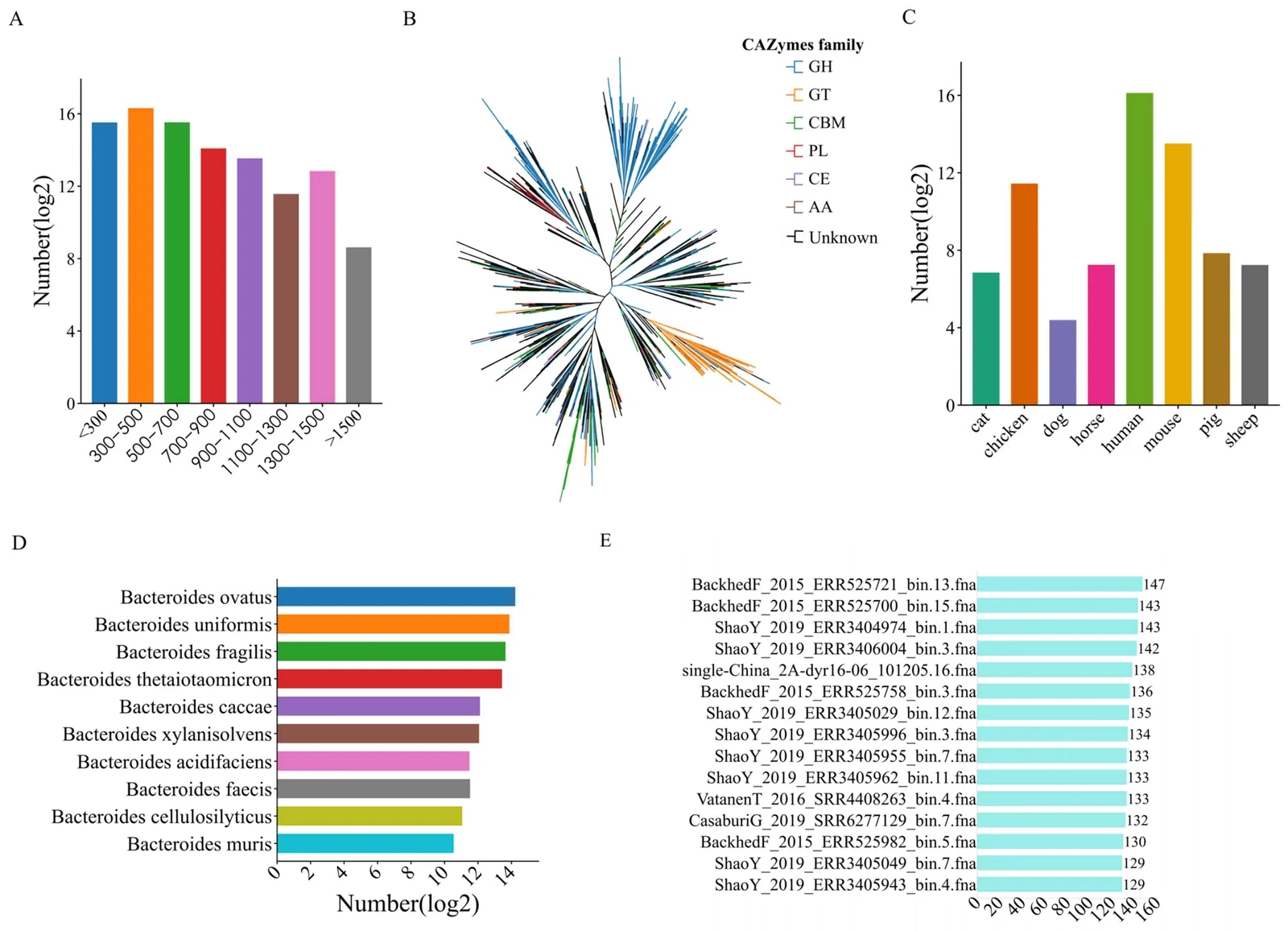
The diversity of potential CAZymes discovered in the *Bacteroides* genus. (**A**) The length distribution of the newly discovered potential novel CAZymes. The x-axis represents the length distribution of potential novel CAZymes, while the y-axis shows the number of potential novel CAZymes of different lengths. (**B**) The potential new types of CAZymes, which might belong to a certain family of CAZymes, and their diverse distribution. (**C**) The sources and distribution quantities of these potential novel CAZymes. (**D**) The top 10 species that encode the most novel CAZymes. (**E**) The top 15 genomes that encode the most potential new CAZymes.

Further studies revealed that the majority of these potential CAZymes amino acids was concentrated between 300 and 500 (Figure 3A). Notably, one protein (ShaoY_2019_ERR3404771_bin.7_k141_8402_54) contains 2195 amino acids and has more than ten folding regions. This protein may be a carbohydrate enzyme with multiple domains (Figure S2). These potential CAZymes were clustered using CD-HIT. At the level of sequence similarity of 90% and coverage of 95%, a total of 5255 clusters were obtained. The phylogenetic analysis of the known CAZymes indicates that these potential CAZymes are distributed across multiple different CAZyme families. Indicating the diversity and novelty of this potential carbohydrate enzyme library (Figure 3B). Analysis of the hosts of these potential CAZymes revealed that 82.5% derived from humans and 13.5% from mice (Figure 3C). This may be related to the large number of MAGs found in both species. The results showed that *Bacteroides ovatus* encodes the most potential CAZymes, far more than other species. This was followed by *Bacteroides uniformis* and *Bacteroides fragilis* (Figure 3D). Interestingly, among the top 15 MAGs encoding the largest number of potential CAZymes, only the fifth, single-China_2A-dyr16-06_101205.16.fna, is not a *Bacteroides ovatus*. The other 14 MAGs all belonged to *Bacteroides ovatus*. In addition, among the top 20 MAGs with the largest number of PULs, only one MAG did not belong to *Bacteroides ovatus* (Figure 3E, Supplementary Table S5). Therefore, *Bacteroides ovatus* is the key strain for the discovery of CAZymes.

### 2.4. Bacteroides Ovatus Was the Gut Microbe Encoding the Most PULs

**Figure 4:**
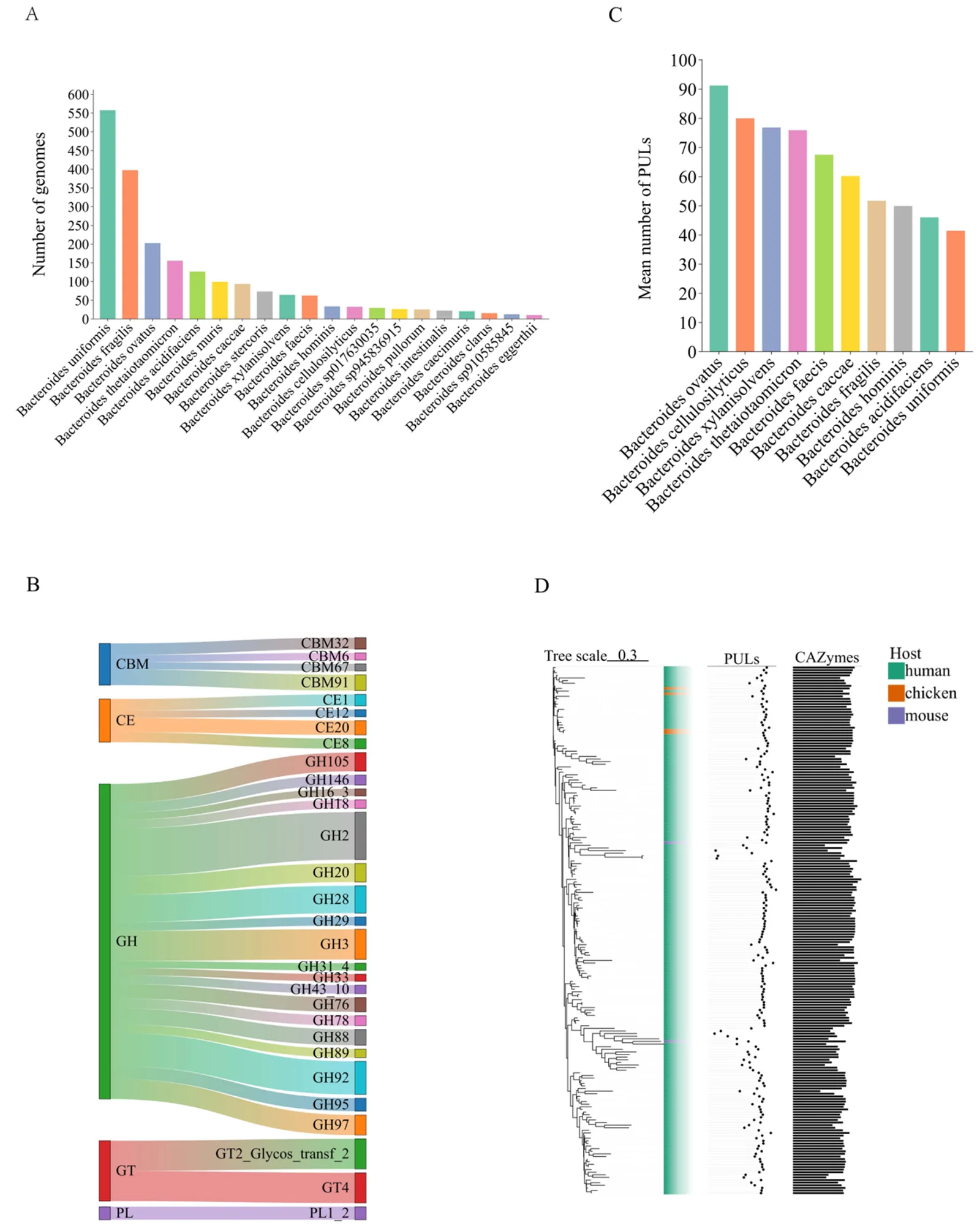
The extremely strong CAZyme-encoding ability of *Bacteroides ovatus*. (**A**) Distribution of the number of individual species in our collection of *Bacteroides*. (**B**) The top 30 CAZyme families with the highest encoding quantity in *Bacteroides uniformis*. (**C**) The average number of PULs encoded by each species in the genus *Bacteroides*. (**D**) Evolutionary relationships of *Bacteroides ovatus* and its strong ability to encode CAZymes and PULs. The first column represents the evolutionary tree of the *Bacteroides ovatus* and its host. The PULs column indicates the number of PULs encoded by the genome. The CAZymes column shows the number of CAZymes enzymes encoded by the genome.

The largest group within the *Bacteroides* genus is *Bacteroides uniformis* (25.0%), followed by *Bacteroides fragilis* (17.8%) and *Bacteroides ovatus* (9.1%) (Figure 4A). The GH3 and GH2 families were enriched in *Bacteroides uniformis* (Figure 4B). We found that the average number of CAZymes in *Bacteroides ovatus* was 1.67 times higher than in *Bacteroides uniformis* (Figure 4C).

Among the 2228 MAGs of the *Bacteroides* genus, 64 of them were able to encode at least 100 PULs. Interestingly, 58 MAGs are *Bacteroides ovatus*, and Vatanent_2016_SRR4408032_bin.1 has the most PULs (119 PULs). These results once again emphasize the extremely important role of *Bacteroides ovatus* in the degradation of carbohydrates in the animal gut. Phylogenetic tree analysis showed that the *Bacteroides ovatus* strains from different animals did not cluster together. Conversely, strains with a similar number of encoded PULs and CAZymes were found to be closer.

### 2.5. Verification of the Potential CAZymes Library

**Figure 5:**
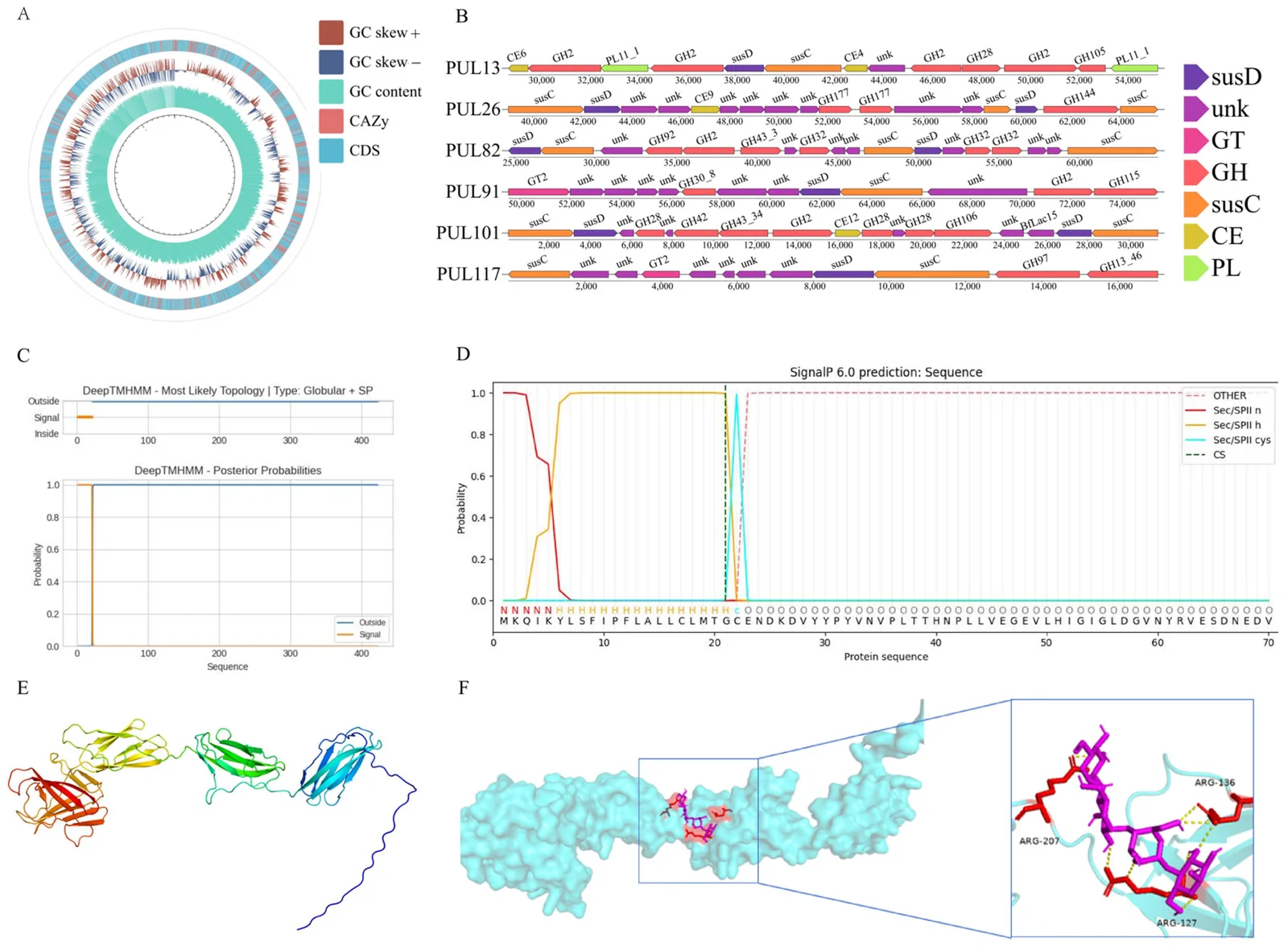
Screening of a potential new CAZyme with the lowest feasibility from Vatanent_2016_SRR4408032_bin.1. (**A**) Map of the genome circle of Vatanent_2016_SRR4408032_bin.1. (**B**) PULs encoded in Vatanent_2016_SRR4408032_bin.1 containing a large number of known CAZymes and potentially novel CAZymes. (**C**) Transmembrane structure prediction of *BfLac15*. (**D**) Signal peptide prediction for *BfLac15*. (**E**) Three-dimensional structure of *BfLac15*. (**F**) Visualization of molecular docking of *BfLac15* with maltotetraose.

To verify the reliability of this potential CAZymes library, we randomly selected Vatanent_2016_SRR4408032_bin.1 for further validation. This MAG encodes 5042 coding sequence (CDS) fragments, 640 of which are annotated as CAZymes. Its PULs contain 102 potential CAZymes (Figure 5A,B). To efficiently evaluate the activity of potential CAZymes, we selected the protein with the lowest probability from these 102 potential CAZymes for further verification. Vatanent_2016_SRR4408032_bin.1_k119_47777_15 was ultimately selected for verification. It has no known CAZyme domain and has the lowest sequence similarity (24.7%). The 3D structure of *BfLac15* was constructed using expasy, with a confidence score GMQE of 0.83, which is of high credibility (Supplementary Table S8). For identification purposes, we named it *BfLac15* (Figure 5B). It is located at the end of PUL101, close to the SusC/D structural region. PUL101 encodes a total of 17 proteins, including two groups of SusC/D structures. It also encodes seven CAZymes from the GH family and one CAZyme from the CE family.

The physicochemical properties of *BfLac15* were predicted. *BfLac15* is composed of 423 amino acids. Its predicted molecular weight is 46.47 kDa, its predicted isoelectric point is 4.75, and its predicted instability index (II) is 24.28. It is a stable protein. The grand average of hydrophobicity is −0.256, indicating weak hydrophilicity. At the N-terminus of *BfLac15*, extremely strong hydrophilicity is observed (Figure S3A). Prediction of signal peptides shows that the Sec/SPII tag from 1–21 strongly suggests the presence of a signal peptide (Figure 5C,D). Based on the transmembrane domain prediction, we speculate that this protein will be secreted outside the cell and may be anchored to the cell membrane. The 3D structure of *BfLac15* revealed four distinct structures, suggesting that *BfLac15* may be a multifunctional carbohydrate enzyme (Figure 5E). Predicted phosphorylation sites revealed that *BfLac15* contains a large number of potential sites; for example, Y326, Y328 and Y373 are potential phosphorylation sites (Figure S3B).

We conducted a docking experiment involving the *BfLac15* protein and nine types of small-molecule carbohydrate. *BfLac15* acted as the receptor, while the carbohydrates acted as the ligands. Of these, the lowest binding energy was found with maltotetraose, at −5.688 (Figure 5F). The lower the binding energy, the more stable the binding of the protein to the substrate. The next lowest binding energy was found with xylan, at −5.549 (Figure S3C). Maltose and amylopectin follow, with values of −5.204 (Figure S3D) and −5.162 (Figure S3E), respectively. Of these nine small molecules, only CMC-Na has a binding energy greater than −4 with *BfLac15*, and this binding is unstable [17]. The bindings of the other eight to *BfLac15* are all relatively stable, which suggests that it may be a novel multifunctional carbohydrate enzyme. 

### 2.6. Prokaryotic Expression of BfLac15 and Determination of Its Physicochemical Properties

**Figure 6:**
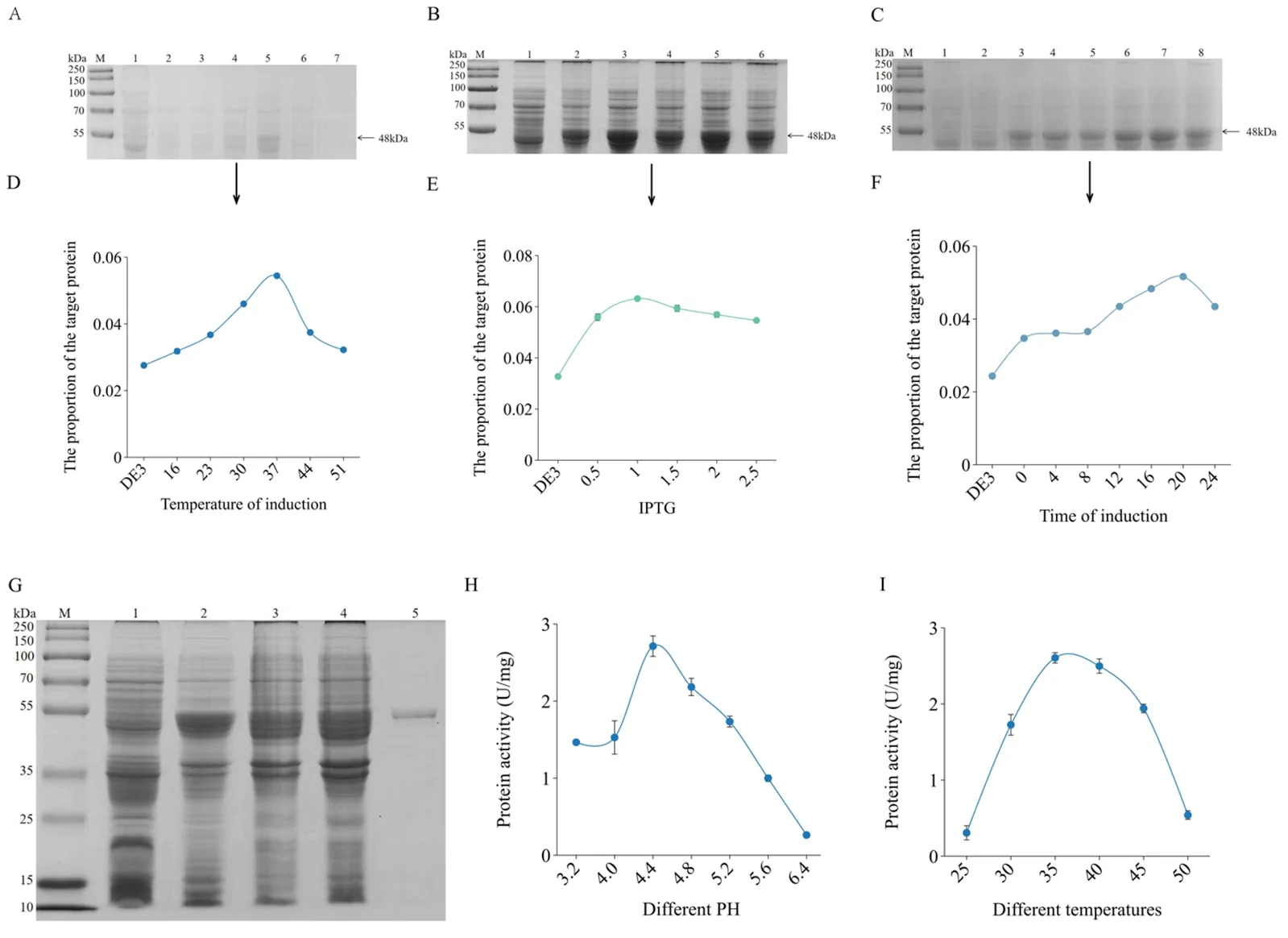
Prokaryotic expression and enzymatic characterization of *BfLac15*. (**A**) Effect of different induction temperatures on the expression of *BfLac15*. M—Protein molecular weight standards; line 1—Total content of target protein without inducer; line 2–7—Total protein expression induced by 16 °C–51 °C. (**B**) Visualization of target proteins induced by different IPTG concentrations. M—Protein molecular weight standards; line 1—Total content of target protein without inducer; line 2–6—Total protein expression induced by 0.05–0.25% IPTG. (**C**) Visualization of target proteins at different induction times. M—Protein molecular weight standards; line 1—Total content of target protein without inducer; line 2–8—The content of target protein at 0–24 h induced by IPTG. Each interval is 4 h. (**D**) The proportion of the target protein in the total protein at different induction temperatures. (**E**) The proportion of the target protein in the total protein under different concentrations of inducers. (**F**) The proportion of the target protein in the total protein at different induction times. (**G**) Expression and purification of *BfLac15*. M—Protein molecular weight standards; line 1—Total protein of uninduced cells; line 2—Total protein of induced cells; line 3—Total protein was induced in the supernatants of sonicated cells; line 4—The total protein in the precipitate after sonication of the cells was induced; line 5—protein washed with 250 mM imidazole buffer. (**H**) Determination of the optimal pH for carbohydrate degradation by *BfLac15*. (**I**) Determination of the optimum temperature for carbohydrate degradation by *BfLac15*.

To verify the function of *BfLac15*, we constructed the PET-28a^+^-*BfLac15* recombinant vector and transformed it into the DE3 strain to verify its expression. SDS-PAGE analysis revealed that the positive strains induced by IPTG exhibited the expected bands. To enhance expression, culture conditions were optimized by adjusting induction time, temperature and IPTG concentration gradients. The results showed that the proportion of *BfLac15* in the total protein reached the highest value when the induction temperature was 37 °C (Figure 6A,D), the IPTG concentration was 1.0 mmol/L (Figure 6B,E) and the induction lasted for 20 h (Figure 6C,F), and this protein was expressed in a soluble form (Figure 6G). The *BfLac15* protein was purified using a prepacked HisTrap HP column, and a single protein band of the expected size was obtained (Figure 6G).

The carbohydrate enzymatic activity of the *BfLac15* protein was determined using the DNS assay. Of the 19 substrate species tested, *BfLac15* demonstrated the greatest capacity for degrading xylan (3.24 U/mg) and β-glucan (2.63 U/mg) (Supplementary Table S9). There is no degradation activity for the other 17 types of small molecules. The physicochemical properties of *BfLac15* were investigated using β-glucan as the substrate, given the poor solubility of xylan and the good solubility of β-glucan. The enzymatic activity of *BfLac15* was then tested under different temperature, pH and metal ion conditions. The results showed that the enzyme activity of *BfLac15* peaked at pH = 4.4 (Figure 6H). At pH = 4.4, the optimum reaction temperature for *BfLac15* was found to be 35 °C (Figure 6I). Furthermore, to evaluate the effect of metal ions on *BfLac15*, enzyme activity was evaluated by testing 10 metal ions, including Mg^2+^, Cu^2+^, Cu^+^, Fe^2+^, Na^+^, Zn^2^^+^, K^+^, Ca^2+^, Mn^2+^ and Fe^3+^, at a final concentration of 5 mmol/L. Zn^2+^, K^+^, Cu^2+^ and Ca^2+^ can inhibit *BfLac15* activity, while Cu^+^, Fe^2+^ and Fe^3+^ can inactivate it (Supplementary Table S10). These results indicate *BfLac15* is a novel multifunctional carbohydrate enzyme. This further confirms the high potential of our discovered carbohydrate enzyme library.

### 2.7. Expansion of BfLac15 Families

**Figure 7:**
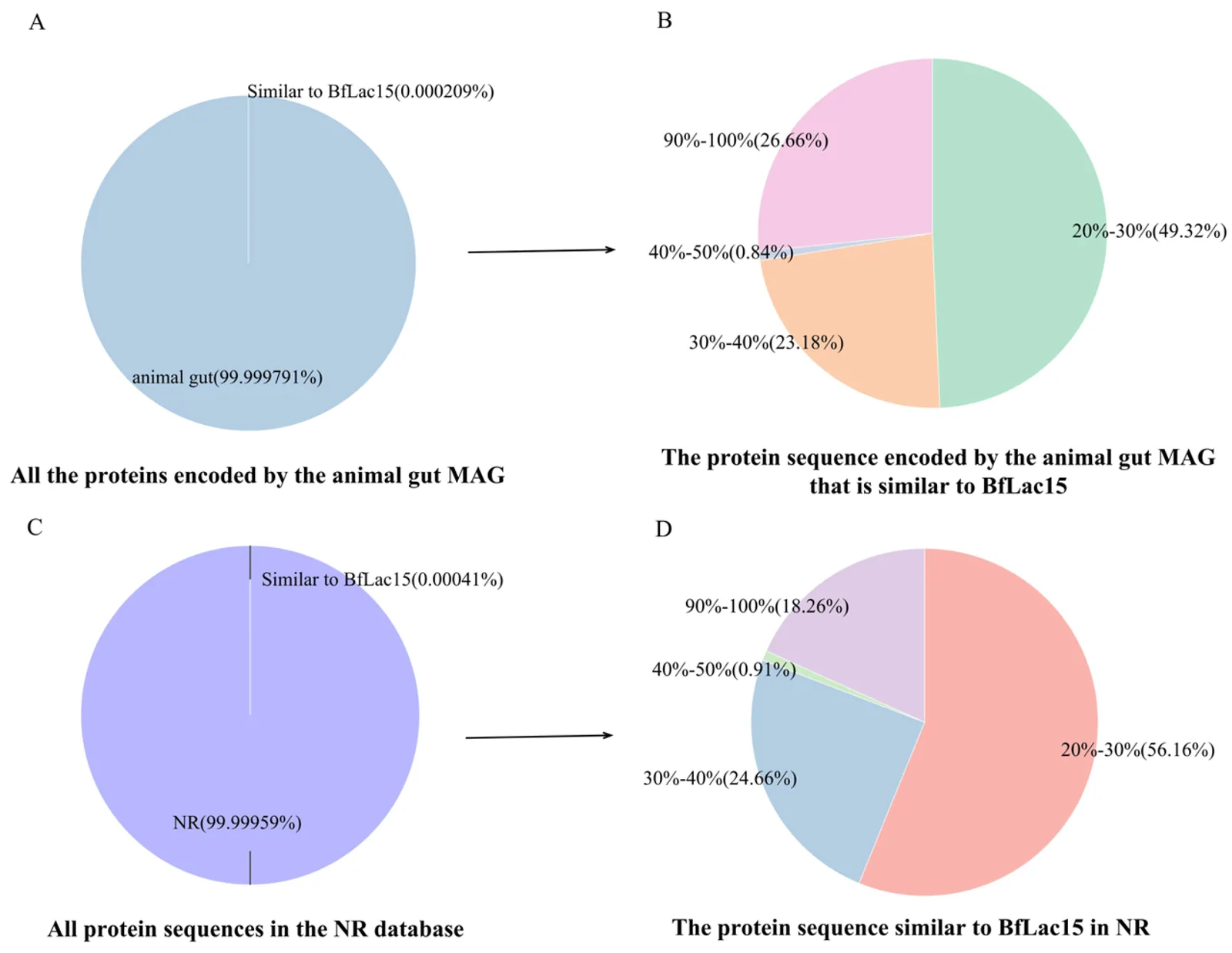
Expansion of the CAZyme family of *BfLac15*. (**A**) The proportion of CAZymes that are similar to *BfLac15* found in the intestinal tracts of animals. (**B**) The similarity distribution of CAZymes found in the intestines of animals that are similar to *BfLac15*. (**C**) The proportion of CAZymes in the NR library that have a 20% similarity to *BfLac15*. (**D**) The distribution of similarities found in the NR database for CAZymes that are similar to *BfLac15*.

We used the BLASTp method to compare *BfLac15* with the proteins encoded by animal gut MAGs and the proteins in the NR database to expand the potential *BfLac15* family. Of the animal gut MAGs, 255 proteins showed over 90% similarity (Figure 7A,B), while the NR database contained 79 proteins with over 90% identity (Figure 7C,D). However, no protein sequence with moderate similarity (50–90%) to *BfLac15* was found in these two libraries, suggesting that *BfLac15* may be a relatively conserved CAZyme. Additionally, we identified 481 protein sequences in the animal gut that share more over 30% similarity with *BfLac15*, Of these, 376 protein sequences can be found in the potential CAZyme library, accounting for 83.4%. The remaining 75 sequences are likely encoded in non-PULs (Figure S4A,B). This further validates the reliability of the potential CAZyme library and provides a new approach for identifying novel CAZymes encoded in non-PULs. Animal gut-encoded sequences with more than 30% similarity to *BfLac15* were extracted, and the same protein sequences were removed using CD-HIT. This resulted in 75 protein sequences for which a phylogenetic tree was constructed. None of the 75 proteins were annotated using run_dbcan2 (Figure S4C). This suggests that *BfLac15* may belong to a new family of CAZymes.

## 3. Discussion

Carbohydrates (including monosaccharides, oligosaccharides, polysaccharides and their derivatives) are substances that are abundant in nature. They are not only the primary energy source for humans and animals, but also the raw materials used to produce food, medicine, materials and chemicals [18,19,20]. The gut microbiota constitutes a vast reservoir of CAZymes and plays a crucial role in carbohydrate research. For example, short-chain fatty acids (SCFAs), primarily secreted by gut microorganisms, exhibit anti-inflammatory properties and can modulate epigenetic remodeling and influence host metabolism [21]. Gut microbes degrade carbohydrates for their own growth and return the energy they receive to the host in the form of SCFAs, typically accounting for 10% of the host’s energy budget [22]. Thus, animals and gut microbes are in a mutually beneficial relationship [23]. Additionally, G Chihara reviewed the use of β-1,3-glucanase to modify lentinan polysaccharide and enhance its ability to activate macrophages [24]. These enzymes, which are derived from the body’s own carbohydrates, can be used to prevent and treat various diseases and to eliminate the possible effects of foreign proteins on the host. Therefore, studying CAZymes in animal gut microbiota advances our understanding of complex carbohydrate metabolism and provides valuable resources for the developing of new drugs.

In this study, by integrating 100,090 high-quality MAGs reconstructed from the gut microbiomes of 11 animal species with distinct feeding habits, this work systematically delineated the global landscape of gut microbial CAZymes and further established a targeted mining pipeline for novel carbohydrate-degrading enzymes centered on *Bacteroides* [22,25]. Our taxonomic profiling showed that Bacteroidota and Bacillota dominate the CAZyme-encoding microbiota, with *Bacteroides* standing out as the genus with the greatest CAZyme repertoire, reinforcing the well-recognized role of *Bacteroides* as the primary glycan-degrading guild in animal intestinal ecosystems [14,26]. Diet-driven functional stratification was clearly reflected in the CAZyme profiles: herbivorous hosts uniquely harbored GH124, GH16_25, GH45, GH45_1, GH54 and GH5_52 families associated with forage polysaccharide breakdown, while omnivores possessed signature families GH62, GH43_23 and GH150, which were absent in carnivorous and herbivorous groups [27]; this supports the hypothesis that host diet acts as a powerful selective filter shaping the accessory CAZyme repertoire of gut microbes [10]. Unexpectedly, the human gut MAGs exhibited the highest average number of CAZymes per genome, even exceeding ruminants, which rely heavily on fibrous plant biomass [9]; this observation echoes recent reports that the omnivorous human gut microbiota maintains an exceptionally flexible glycan-degrading capacity to cope with highly variable dietary polysaccharide inputs rather than a narrow fiber-specialized program [3]. This functional divergence cautions against the simple assumption that herbivorous animals always encode more CAZymes, and highlights that the combinatorial diversity of PULs and CAZymes, rather than host trophic category alone, determines glycan utilization potential [6].

Among all recovered *Bacteroides* MAGs, we identified 2228 medium- and high-quality genomes with low contamination, and *Bacteroides ovatus* emerged as the key reservoir of PULs and CAZymes. *B. ovatus* encoded far more PULs and CAZymes than closely related species such as *Bacteroides uniformis* and *Bacteroides fragilis*, and nearly all MAGs carrying >100 PULs belonged to *B. ovatus*. This finding shows that *B. ovatus* has evolved an expanded, substrate-tunable PUL arsenal to target a broad spectrum of plant, host and algae-derived glycans, including xylan, β-glucan, alginate and pectin [28,29]. The SusC protein was the most abundant annotated component in the Kofamscan prediction, which is consistent with the canonical Bacteroidete glycan-import paradigm: SusC/SusD outer-membrane transporter pairs capture oligosaccharides generated by cell-surface CAZymes and deliver them into the periplasm for further processing by GH, PL and CE enzymes encoded within the same PUL [30,31]. Our phylogenetic reconstruction showed that *Bacteroides* strains from different animal hosts did not strictly cluster by host origin; instead, strains with similar PUL and CAZyme loads tended to group together, indicating that horizontal gene transfer and PUL expansion, rather than host co-speciation, are major forces driving the evolution of carbohydrate catabolism in this genus [32].

Traditional CAZyme annotation pipelines relying solely on HMM homology frequently miss novel divergent enzymes, which severely limits the discovery of new biocatalysts from metagenomic dark matter [33]. To overcome this bottleneck, we combined homology filtering, length screening and structure-aware deep-learning prediction using DeepFRI to prioritize candidate CAZymes from hundreds of thousands of hypothetical proteins within PULs. This multi-layered strategy yielded a library of 90,286 potential novel CAZymes, the majority of which were contributed by human-derived *Bacteroides* MAGs, especially *B. ovatus*. Structure-based functional predictors such as DeepFRI complement sequence homology searches by capturing fold and active-site geometric features [34], and have been increasingly adopted for mining metagenomic enzyme candidates with low sequence identity to characterized CAZymes [35]. The selected representative enzyme *BfLac15* had no recognizable canonical CAZyme domain and only 24.7% identity to known sequences, yet molecular docking indicated favorable binding to maltotetraose, xylan, maltotriose and amylopectin, implying a broad substrate range. Subsequent heterologous expression and DNS activity assays validated that *BfLac15* preferentially degrades xylan and β-glucan under mildly acidic conditions, confirming it as a bona fide bifunctional novel CAZyme [36]. This experimental validation demonstrates that our AI-assisted screening workflow is effective for unlocking functional enzymes from PUL-encoded hypothetical proteins that escape standard dbCAN annotation.

Family expansion analysis further supports the finding that *BfLac15* represents a founding member of a previously uncharacterized CAZyme family. We recovered hundreds of homologues sharing >30% sequence identity, and none of these expanded sequences received confident family assignments by run_dbcan2, whereas high-identity homologues (>90%) were sparsely distributed and showed limited intermediate similarity (50–90%), consistent with a conserved, deeply divergent enzyme lineage [37]. Notably, 16.6% of *BfLac15*-like sequences were located outside annotated PUL regions, suggesting that novel CAZymes are not exclusively confined within classical PUL architectures in *Bacteroides* [5]. This expands the current view that PUL-independent glycan-degrading genes also contribute to the carbohydrate catabolic network, and future mining efforts should not be restricted to annotated PUL boundaries [38]. From an applied perspective, novel bifunctional enzymes active on xylan and β-glucan are highly desirable for biorefinery, feed and fine-chemical industries [39], because they can simultaneously target two abundant components of plant cell walls and reduce the number of enzyme preparations required for lignocellulose saccharification [7].

In summary, this large-scale MAG-based study establishes that *B. ovatus* is an unparalleled reservoir of novel CAZymes in the animal gut. The integration of PUL mining, structure-based deep-learning prediction and in vitro biochemical validation provides a scalable framework for discovering divergent carbohydrate-active enzymes from metagenomic dark matter. The characterized *BfLac15* represents a promising bifunctional biocatalyst, and the constructed candidate library lays a foundation for further screening of robust enzymes to support lignocellulose bioconversion, alleviate energy-resource pressure and expand the biobased fine-chemical industry chain [40]. Future work will focus on high-throughput expression of prioritized candidates, engineering of thermostability and specific activity, and evaluation of their performance in biomass pretreatment and oligosaccharide production.

## 4. Materials and Methods

### 4.1. Experimental Materials

3,5-Dinitrosalicylic Acid (Solarbio, Beijing, China), Isopropyl β-D-Thiogalactopyranoside (IPTG, Purity ≥ 99.00%, Solarbio, Beijing, China), Tris (Hydroxymethyl) Aminomethane (tris, Purity ≥ 99.5%, Solarbio, Beijing, China), NaCl (Purity ≥ 99.5%, Sangon Biotechnology, Shanghai, China), 10% SDS Solution (Sangon Biotechnology, Shanghai, China), Ammonium persulfate (APS, Purity ≥ 98.5%, Sangon Biotechnology, Shanghai, China), N,N,N′,N′-Tetramethylethylenediamine (TEMED, Purity ≥ 99.0%, Sangon Biotechnology, Shanghai, China), BCA kit (Solarbio, Beijing, China), PBS buffer (Sangon Biotechnology, Shanghai, China).

Polysaccharide substrate include carboxymethyl cellulose (MW 354.375 g/mol, Purity ≥ 99.5%, DS = 0.89, Sangon Biotechnology, Shanghai, China), xylan (MW (132.11×n) g/mol, Purity ≥ 90%, Source leaf, Shanghai, China), raffinose (MW 594.51 g/mol, Purity ≥ 98%, Solarbio, Beijing, China), sucrose (MW 342.29 g/mol, Purity ≥ 98%, Solarbio, Beijing, China), arabinose (MW 150.13 g/mol, Purity ≥ 98%, Solarbio, Beijing, China), Sodium hyaluronate (MW (401.3×n) g/mol, Purity ≥ 90%, Source leaf, Shanghai, China), lactose (MW 150.13 g/mol, Purity ≥ 99%, Solarbio, Beijing, China), D-(+)-trehalose dihydrate (MW 378.33 g/mol, Purity ≥ 98%, Solarbio, Beijing, China), amylose (MW (162.14×n) g/mol, Purity ≥ 95%, Source leaf, Shanghai, China), melibiose (MW 360.31 g/mol, Purity ≥ 98%, Solarbio, Beijing, China), Maltotriose (MW 504.44 g/mol, Purity ≥ 98%, Solarbio, Beijing, China), D-(+)-maltose monohydrate (MW 360.31 g/mol, Purity ≥ 98%, Solarbio, Beijing, China), D-mannose (MW 180.16 g/mol, Purity ≥ 98%, Solarbio, Beijing, China), D-salicin (MW 286.28 g/mol, Purity ≥ 98%, Solarbio, Beijing, China), D-(+)-cellobiose (MW 342.3 g/mol, Purity ≥ 98%, Solarbio, Beijing, China), β-glucan (MW (162.14×n) g/mol, Purity ≥ 95%, source leaf, Shanghai, China), polysucrose (Ficoll 400, Purity ≥ 99%, Solarbio, Beijing, China), amylopectin (MW (162.14×n) g/mol, Purity ≥ 95%, source leaf, Shanghai, China), arbutin (MW 272.25 g/mol, Purity ≥ 98%, source leaf, Shanghai, China).

### 4.2. Species Distribution in the Gut of Different Animals

The dataset was compiled from a total of 100,090 metagenome-assembled genomes (MAGs) harvested from the intestinal microbiomes of diverse animal hosts, covering donkeys (6684 MAGs) [11], cats (112 MAGs) [41], chickens (18,688 MAGs) [42,43,44], cattle (1467 MAGs) [45,46], horses (4247 MAGs) [47,48], dogs (34 MAGs) [49], ferrets (26 MAGs) [50], humans (32,277 MAGs) [51], mice (23,364 MAGs) [52,53,54,55], pigs (7381 MAGs) [56], and sheep (5810 MAGs) [57]. Taxonomic classification and annotation of all collected MAGs were performed using GTDB-Tk (version 2.4.0) [58].

### 4.3. Distribution of CAZymes in Different Bacterial Genera

To further compare the differences in CAZymes between species, run_dbcan2 [59] was used to annotate CAZymes for all MAGs. In addition, the genus encoding the most CAZymes was selected and the PhyloPhlAn 3.0 [60] software was used to construct the proteomic phylogenetic tree of these bacteria (-f supermatrix_aa.cfg --diversity medium --fast). Genome quality was assessed with the use of checkM2 (v 1.1.0) [61]. KEGG annotation of the genus with the most CAZymes was performed using kofamscan [62].

### 4.4. To Explore Potential New CAZymes

To further explore potential novel efficient CAZymes, PULs were predicted using PULpy [63,64], and potential novel efficient CAZymes were expected to be mined from PULs. The annotation results of PULpy and run_dbcan2 were combined to extract the unannotated protein sequences. Using the AI model DeepFRI [65], these unknown proteins were further annotated to remove the non-CAZymes that are encoded on the PULs. Currently, among the known CAZymes, the smallest one is CelOCE, which is one of the smallest catalytically active proteins discovered in carbohydrate enzymology, containing only 115 amino acids [57]. Therefore, protein sequences of less than 115 amino acids were removed. The remainder were integrated into the library of potential CAZymes; redundant protein sequences in the library of potential CAZymes were removed with CD-HIT (v 4.8.1). Small differences can lead to large changes in protein function; therefore, strict screening parameters (-c 0.9 -n 5 -M 16000 -T 50 -aS 0.9) were selected [35]. Protein sequences were aligned using MAFFT, and then a phylogenetic tree of potential CAZymes was constructed using IQ-TREE2 (-m MFP) [66]. To judge which class these potential CAZymes belong to, we used BLASTp to align the clustered sequences against a library of known CAZymes with a threshold of 30% [57].

### 4.5. Validation of Potential Novel Carbohydrate Enzyme Libraries

To verify the accuracy of the potential carbohydrate enzyme library, the genomic coding potential CAZymes containing the most PULs were extracted and compared with the CAZymes library by BLASTp (v 2.16.0+), and the sequences with 10% to 30% sequence similarity were selected as candidate enzymes. These candidate enzymes were analyzed by homology modeling (https://swissmodel.expasy.org/, accessed on 8 July 2025) [67], and the candidate enzyme with the lowest confidence was selected for verification.

### 4.6. Predict the Potential Enzymatic Activity of Carbohydrates

A variety of carbohydrate substrates were downloaded from PubChem (https://pubchem.ncbi.nlm.nih.gov/, accessed on 15 July 2025) and the RCSB Protein Database (RCSB) (https://www.rcsb.org/, accessed on 15 July 2025). Carbohydrate small molecules were converted to PDB format using openbabel (v 3.1.1) [68], carbohydrate small molecules and candidate proteins were converted to PDBQT format using AutoDock 4.2.6, carbohydrate small molecules were used as ligands and candidate proteins as receptors, and molecular docking was performed using AutoDock Vina [69]. The docking results were visualized using PyMOL (v 3.1.4.1) [70]. The secondary and tertiary structures of the proteins were predicted using the Swiss-Model servers (http.//web.expasy.org, accessed on 30 July 2025). Theoretical molecular weight (Mw) and isoelectric point (pI) were estimated using ProtParam (http.//web.expasy.org/protparam, accessed on 30 July 2025) [71]. The water solubility of the amino acid sequences was analyzed using the ProtScale online tool [57]. Furthermore, the presence of an N-terminal signal peptide was predicted using SignalP 6.0 [72], and transmembrane domains were identified using DeepTMHMM–1.0 [73]. NetPhos 3.1 (https://services.healthtech.dtu.dk/services/NetPhos-3.1/, accessed on 30 July 2025) was used to predict the phosphorylation sites of candidate proteins [74].

### 4.7. Determining the Optimal Conditions for Producing Enzymes

The gene containing the candidate protein was synthesized by Sangon Biotech Co, Ltd., and the recombinant vector was constructed using PET-28a^+^ plasmid (Shanghai, China). *E. coli* DH5α cells carrying the recombinant plasmid were inoculated into the LB liquid medium supplemented with kanamycin sulfate, and were cultured overnight at 37 °C and 180 rpm. Then, the plasmid was extracted using a plasmid extraction kit (Sangon Biotech, Shanghai, China). The purified plasmid was sent to Sangon Biotech for sequencing and verification. The verified recombinant plasmid was transformed into *E. coli* DE3 for protein expression [16].

In order to explore the best expression conditions of candidate proteins, different induction temperatures (16 °C, 23 °C, 30 °C, 37 °C, 44 °C and 51 °C) were used to induce the expression of candidate proteins. At the optimal induction temperature, different IPTG concentration gradients (0.5‰, 1‰, 1.5‰, 2‰, 2.5‰) were set to determine the optimal inducer concentration. Under the optimal induction temperature and the optimal concentration of IPTG, the experiments were carried out at different induction times (0 h, 4 h, 8 h, 12 h, 16 h, 20 h and 24 h). SDS-PAGE analysis was performed on a 12% concentration gradient gel. DE3 transfected into an empty vector was used as a negative control.

### 4.8. Expression and Purification of Candidate Proteins

The recombinant plasmids were transformed into *E. coli* BL21(DE3) competent cells. Single positive transformants were picked and cultured in LB medium supplemented with corresponding antibiotics at 37 °C, including shaking at 220 rpm until the optical density at 600 nm (OD_600_) reached 0.6–0.8. Protein expression was induced by the addition of IPTG at the predetermined optimal concentration, followed by incubation for 16 h at 18 °C with shaking at 200 rpm [75]. The induced bacterial pellets were harvested by centrifugation at 8000 rpm for 10 min at 4 °C. Cell pellets were resuspended in 30 mL lysis buffer (50 mM Tris–HCl, 500 mM NaCl, pH 7.5) and incubated overnight at 4 °C. The cell suspension was subjected to three rounds of freeze–thaw cycling using liquid nitrogen and a 37 °C water bath, followed by sonication at 90 W on ice to disrupt cells. After sonication, the lysate was centrifuged at 8000 rpm for 10 min at 4 °C. The resulting supernatant was collected and filtered through a 0.22 μm filter membrane. Target recombinant proteins were purified using a prepacked HisTrap HP column (GE Healthcare Life Sciences, Logan, UT, USA) via Ni^2+^ affinity chromatography. Purified protein fractions were analyzed by SDS-PAGE using a 12% separating gel, and protein bands were visualized by Coomassie brilliant blue staining. The buffer of purified candidate proteins was exchanged into PBS buffer using ultrafiltration for subsequent enzyme activity assays [16].

### 4.9. Enzyme Activity Assay

The concentration of purified protein was determined using the BCA kit according to the manufacturer’s instructions [16]. The enzyme activity of purified protein was determined by the 3,5-Dinitrosalicylic Acid Method (DNS). In short, a standard curve was established by mixing 300 μL solutions of 1 mg/mL, 2 mg/mL, 3 mg/mL, 4 mg/mL and 5 mg/mL reducing sugars with 200 μL ddH_2_O and 500 μL DNS, respectively. Enzyme activity was defined as the amount of enzyme required to convert 1 micromole substrate in 1 min at the optimal temperature as one enzyme activity unit (U). Enzyme activity determination formula: enzyme activity (U) = (c × K × V)/(M × t × 1000). Formula for specific enzyme activity: specific activity (U/mg) = (c × K × V × 1000)/(M × t × m) [76], where c—product concentration (mg/mL); K—dilution coefficient; V—volume of reaction system (mL); t—reaction time (min); M—molar mass of the product (g/mol); m—mass of purified enzyme protein (mg); 1000—unit conversion factor from mol to μmol. The activity of candidate proteins was determined according to the reaction system: candidate proteins 200 μL, 1% carbohydrate 300 μL, 37 °C, 30 min. At the end of the reaction, 500 μL DNS was added to a boiling water bath for 5 min and placed on ice for rapid cooling. The absorbance was measured at 540 nm [77]. The amount of reducing sugar produced by the degradation of the carbohydrates of the candidate proteins can be determined from the reducing sugar standard curve.

### 4.10. Polysaccharide Hydrolysis Experiment

In the polysaccharide hydrolysis experiment, the following polysaccharide substrates were selected: carboxymethyl cellulose, xylan, raffinose, sucrose, arabinose, sodium hyaluronate, lactose, D-(+)-trehalose dihydrate, amylose, melibiose, maltotriose, D-(+)-maltose monohydrate, D-salicin, D-(+)-cellobiose, β-glucan, polysucrose, amylopectin, and arbutin. In addition, we collected ten metal ions, namely Mn^2+^, Cu^2+^, Mg^2+^, Fe^2+^, Na^+^, Ca^2+^, Cu^+^, Zn^2+^, Fe^3+^, and K^+^, and put them into carbohydrate solution with a final concentration of 5 mmol/L [16]. Polysaccharide hydrolysis experiments were carried out according to the above method of enzyme activity determination.

### 4.11. Determination of Physicochemical Properties of Candidate Proteins

β-glucan was used as a small molecule substrate. The activity of *BfLac15* was measured using 50 mM sodium citrate buffer with a pH range of 3.2 to 6.4 (3.2, 4.0, 4.4, 4.8, 5.2, 5.6 and 6.4). The enzyme and substrate were incubated at temperatures ranging from 20 °C to 50 °C (20 °C, 25 °C, 30 °C, 35 °C, 40 °C, 45 °C and 50 °C) for 30 min to determine the optimal reaction temperature. The enzyme activity at 37 °C and pH = 4.8 were defined as the control, representing 100% activity [16].

### 4.12. Prediction of Candidate Proteins Family Members

To further understanding of the members of the candidate protein family, we used BLASTp (E-value 10^−5^) to compare the distribution of candidate proteins against proteins encoded by the animal intestinal bacteria MAG and the NR database [78], The sequences with more than 30% similarity were selected for CAZyme annotation with run_dbcan2. CD-HIT was used to remove redundancy, and then IQ-TREE was used to construct phylogenetic trees of candidate protein families [79]. This analysis aimed to identify homologous proteins and provide insights into the evolutionary and functional relationships within the candidate protein family.

### 4.13. Statistical Analysis

Experimental results from enzyme activity assays were averaged over three measurements and analyzed using the Statistical Package for Social Sciences (SPSS v23.0) [80]. All data are presented as mean ± standard deviation (SD) derived from three independent experiments (*n* = 3). Images were beautified using CNSknowall (https://cnsknowall.com/index.html#/HomePage) [81] and chiplot (https://www.chiplot.online/) [82].

## Data Availability

The predicted potential carbohydrate enzyme inventory in this article is stored at Mendeley Data, “BfLac15 raw data”, Mendeley Data, V1, doi: 10.17632/txh665cj82.1).

## Supplementary Materials

The following supporting information can be downloaded at: https://www.mdpi.com/article/10.3390/molecules31213980/s1, Figure S1: The distribution of CAZymes in the gut of different animals. (A) The proportion of different CAZyme families in the gut of different animals. (B) The top 50 CAZyme families encoded in the intestines of all animals. (C) The average number of CAZymes encoded by each genome in the gut of different animals. (D) The number of CAZyme families annotated in the gut of different animals. Figure S2: The 3D structure of the largest potential CAZyme ShaoY_2019_ERR3404771_bin.7_k141_8402_548. Figure S3: Prediction of physicochemical properties of *BfLac15* and visualization of molecular docking. (A) Hydrophilicity/hydrophobicity prediction of *BfLac15*. (B) Phosphorylation site prediction of *BfLac15*. (C) Visualization of molecular docking of *BfLac15* with xylan. (D) Visualization of molecular docking of *BfLac15* with maltotriose. (E) Visualization of molecular docking of *BfLac15* with amylopectin. Figure S4: Distribution and evolutionary relationships of *BfLac15* family expanded sequences. (A) The distribution of sequences similar to *BfLac15* on PUL and non-PUL regions. (B) The proportion of *BfLac15* family extended sequences encoded in PULs and non-PULs. (C) Evolutionary relationship of *BfLac15* family expanded sequences after redundancy removal. Table S1: The distribution of bacteria and archaea in the intestines of different animals. Table S2: Distribution of carbohydrate enzymes in the gut of different animals. Table S3: Quality assessment of Bacteroides. Table S4: Distribution of common carbohydrate enzymes in Bacteroides. Table S5: The number of PUL and the number of carbohydrate enzymes encoded by each Bacteroide. Table S6: PUL distribution of Bacteroides. Table S7: Functional annotation of potential novel CAZymes. Table S8: Candidate novel CAZymes. Table S9: The influence of different metal ions on the activity of *BfLac15*. Table S10: Enzymatic activity of *BfLac15* on a variety of carbohydrates. All substrates used for branch docking were from PubChem, including Compound CID for various carbohydrates, which were amylopectin (439207), raffinose (439242), maltotriose (192826), trehalose (7427), maltotetraose (123966), sucrose (5988), xylan (129539666), maltose (439186), and CMC-Na (131674687).

## Abbreviations

The following abbreviations are used in this manuscript:

CAZymes	carbohydrate-active enzymes
MAGs	metagenome-assembled genomes
PULs	polysaccharide utilization sites
CAZYs	carbohydrate-active enzymes database
GH	glycoside hydrolases
GTs	glycosyl transferases
PLs	polysaccharide lyases
CEs	carbohydrate esterases
AAs	auxiliary enzyme families
CBMs	carbohydrate binding modules
SCFA	short-chain fatty acids
BLASTP	Protein Basic Local Alignment Search Tool
NCBI	National Center for Biotechnology Information
Mw	molecular weight
pI	isoelectric point
ExPASy	Expert Protein Analysis System
DeepTMHMM	Deep Transmembrane Hidden Markov Model
SOPMA	Self-Optimized Prediction Method with Alignment
iMODS	Integrated Modeling and Simulation for Molecular Dynamics
DNA	deoxyribonucleic acid
LB	Luria–Bertani
IPTG	Isopropyl-beta-D-thiogalactopyranoside
BCA	Bicinchoninic Acid Assay
BSA	bovine serum albumin
DNS	3,5-dinitrosalicylic
SPSS	Statistical Package for the Social Sciences
SDS-PAGE	sodium dodecyl sulfate-polyacrylamide gel electrophoresis
NR	New Radio
ML	maximum likelihood
