# Supplementary material for: Mining and Characterization of Carbohydrate-Active Enzymes in MAGs from Animal Gut Microbes
Source: Molecules. 2026 Nov 4;31(21):3980. doi: 10.3390/molecules31213980 (PMC13765263; doi:10.3390/molecules31213980)
Supplement: Supplementary file 1 [file molecules-31-03980-s001.zip › Figure S1.pdf]

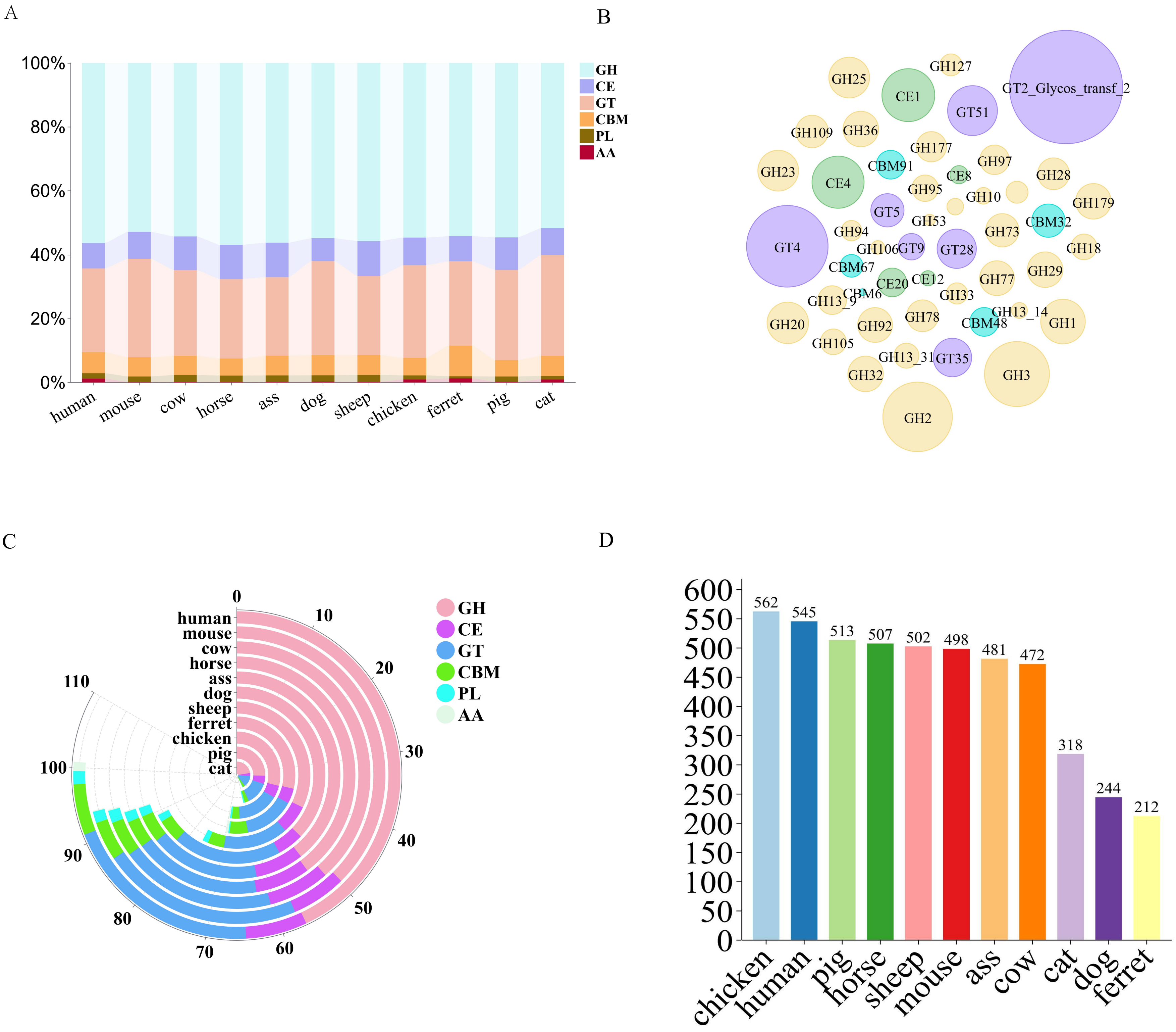

A
100%
80%
60%
40%
20%
0%
GH
CE
GT
CBM
PL
AA
human
mouse
cow
horse
ass
dog
sheep
chicken
ferret
pig
cat
B
GH127
GH25
CE1
GT51
GT2_Glycos_transf_2
GH109
GH36
GH177
GH23
CBM91
GH97
CE4
CE8
GH28
GH95
GH10
GH179
GT5
GH53
GH73
CBM32
GH94
GT4
GH106
GT9
GT28
GH18
CBM67
GH29
CE20
CE12
GH77
CBM6
GH33
GH13_9
GH20
GH92
GH78
CBM48
GH13_14
GH1
GH105
GH13_31
GT35
GH32
GH3
GH2
C
0
10
20
30
40
50
60
70
80
90
100
110
human
mouse
cow
horse
ass
dog
sheep
ferret
chicken
pig
cat
GH
CE
GT
CBM
PL
AA
D
600
550
500
450
400
350
300
250
200
150
100
50
0
562
545
513
507
502
498
481
472
318
244
212
chicken
human
pig
horse
sheep
mouse
ass
cow
cat
dog
ferret
