# Supplementary material for: Mining and Characterization of Carbohydrate-Active Enzymes in MAGs from Animal Gut Microbes
Source: Molecules. 2026 Nov 4;31(21):3980. doi: 10.3390/molecules31213980 (PMC13765263; doi:10.3390/molecules31213980)

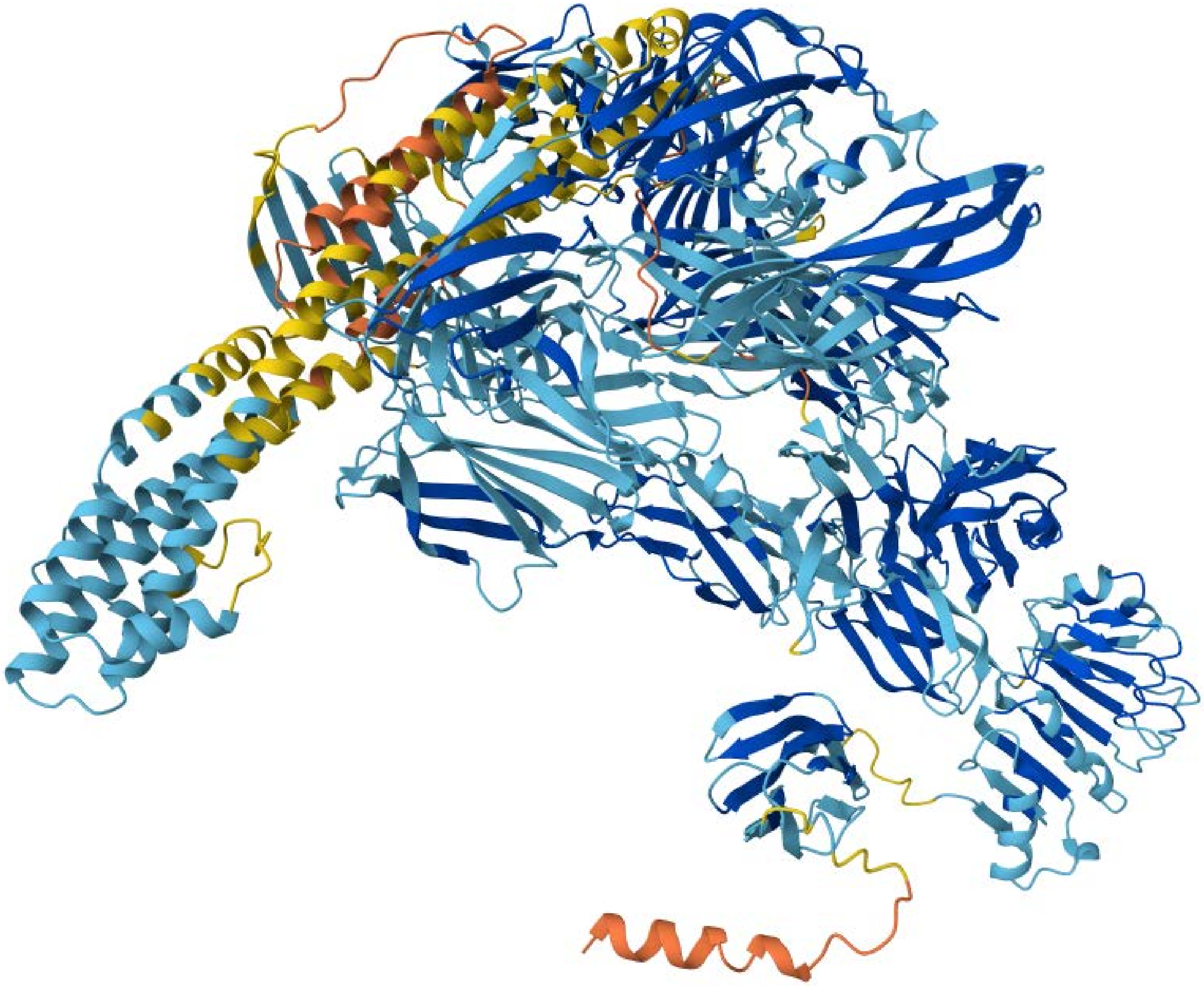

Supplement: Supplementary file 1 [file molecules-31-03980-s001.zip › Figure S2.pdf]
