# Supplementary material for: Mining and Characterization of Carbohydrate-Active Enzymes in MAGs from Animal Gut Microbes
Source: Molecules. 2026 Nov 4;31(21):3980. doi: 10.3390/molecules31213980 (PMC13765263; doi:10.3390/molecules31213980)
Supplement: Supplementary file 1 [file molecules-31-03980-s001.zip › Figure S3.pdf]

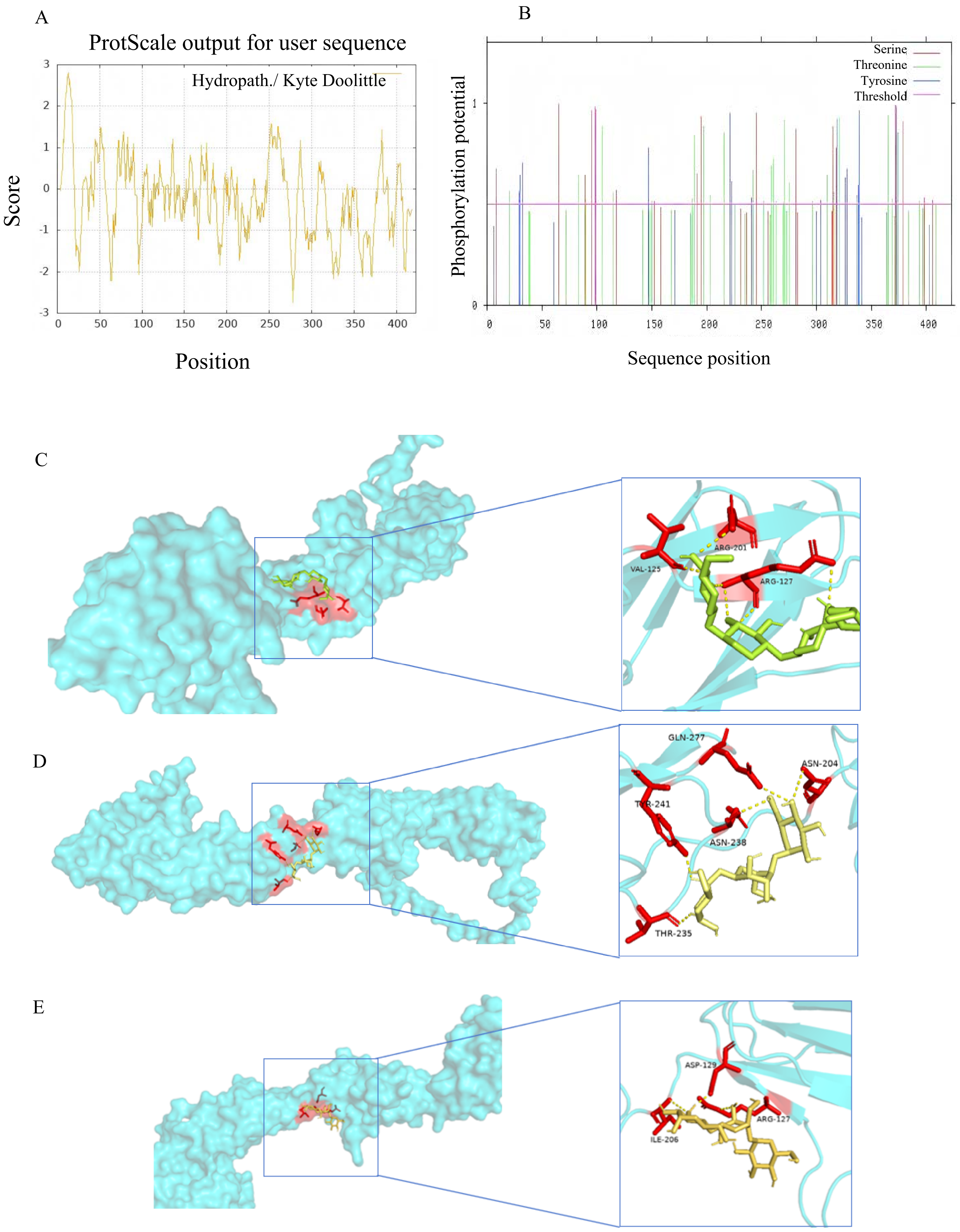

A
ProtScale output for user sequence
Hydropath./ Kyte Doolittle
Score
Position
3
2
1
0
-1
-2
-3
0
50
100
150
200
250
300
350
400
B
Serine
Threonine
Tyrosine
Threshold
Phosphorylation potential
Sequence position
1
0
0
50
100
150
200
250
300
350
400
C
ARG-201
VAL-125
ARG-127
D
GLN-277
ASN-204
ASN-238
THR-235
E
ASP-129
ARG-127
ILE-206
