# Supplementary material for: Mining and Characterization of Carbohydrate-Active Enzymes in MAGs from Animal Gut Microbes
Source: Molecules. 2026 Nov 4;31(21):3980. doi: 10.3390/molecules31213980 (PMC13765263; doi:10.3390/molecules31213980)
Supplement: Supplementary file 1 [file molecules-31-03980-s001.zip › Figure S4.pdf]

A

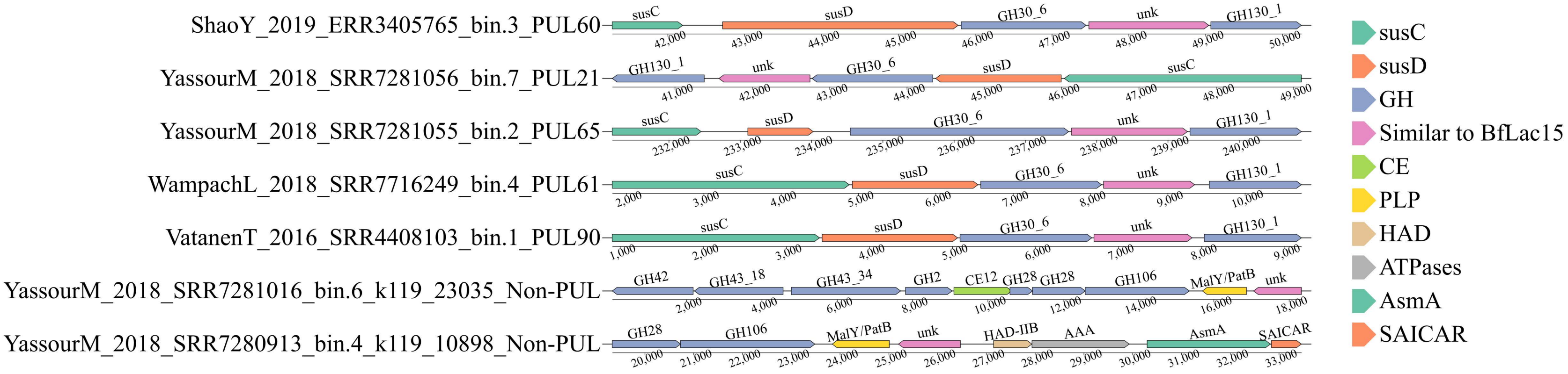
ShaoY_2019_ERR3405765_bin.3_PUL60
susC susD GH30_6 unk GH130_1
YassourM_2018_SRR7281056_bin.7_PUL21
GH130_1 unk GH30_6 susD susC
YassourM_2018_SRR7281055_bin.2_PUL65
susC susD GH30_6 unk GH130_1
WampachL_2018_SRR7716249_bin.4_PUL61
susC susD GH30_6 unk GH130_1
VatanenT_2016_SRR4408103_bin.1_PUL90
susC susD GH30_6 unk GH130_1
YassourM_2018_SRR7281016_bin.6_k119_23035_Non-PUL
GH42 GH43_18 GH43_34 GH2 CE12 GH28 GH28 GH106 MalY/PatB unk
YassourM_2018_SRR7280913_bin.4_k119_10898_Non-PUL
GH28 GH106 MalY/PatB unk HAD-IIB AAA AsmA SAICAR
susC
susD
GH
Similar to BfLac15
CE
PLP
HAD
ATPases
AsmA
SAICAR

B

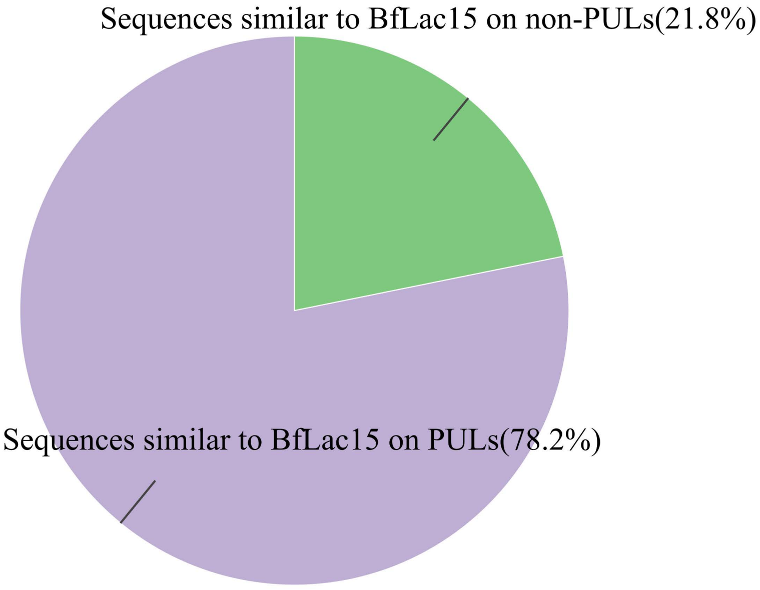
Sequences similar to BfLac15 on non-PULs(21.8%)
Sequences similar to BfLac15 on PULs(78.2%)

C

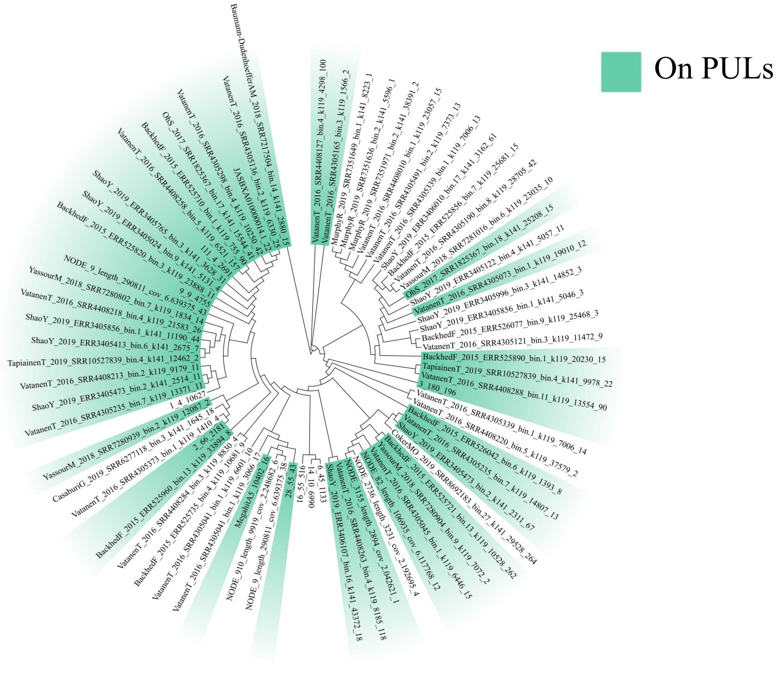
On PULs
